# Exploring factors affecting the unsafe behavior of health care workers’ in using respiratory masks during COVID-19 pandemic in Iran: a qualitative study

**DOI:** 10.1186/s12913-024-11000-4

**Published:** 2024-05-09

**Authors:** Azadeh Tahernejad, Sanaz Sohrabizadeh, Somayeh Tahernejad

**Affiliations:** 1 Department of Health in Disasters and Emergencies, School of Public Health and Safety, Shahid Beheshti University of Medical Sciences, Tehran, 1983535511 Iran; 2 Department of Occupational Health Engineering and Safety at Work, School of Public Health, Kerman University of Medical Sciences, Kerman, Iran

**Keywords:** Respiratory mask, COVID-19, Pandemic, Health care workers

## Abstract

**Background:**

The use of respiratory masks has been one of the most important measures to prevent the spread of COVID-19 among health care workers during the COVID-19 pandemic. Therefore, correct and safe use of breathing masks is vital. The purpose of this study was to exploring factors affecting the unsafe behavior of health care workers’ in using respiratory masks during the COVID-19 pandemic in Iran.

**Methods:**

This study was carried out using the conventional qualitative content analysis. Participants were the number of 26 health care workers selected by purposive sampling method. Data collection was conducted through in-depth semi-structured interviews. Data analysis was done using the content analysis approach of Graneheim and Lundman. This study aligns with the Consolidated Criteria for Reporting Qualitative Research (COREQ) checklist and was conducted between December 2021 and April 2022.

**Results:**

The factors affecting the unsafe behavior of health care workers while using respiratory masks were divided into 3 main categories and 8 sub-categories. Categories included discomfort and pain (four sub-categories of headache and dizziness, skin discomfort, respiratory discomfort, feeling hot and thirsty), negative effect on performance (four sub-categories of effect on physical function, effect on cognitive function, system function vision, and hearing), and a negative effect on the mental state (two subcategories of anxiety and depression).

**Conclusion:**

The findings can help identify and analyze possible scenarios to reduce unsafe behaviors at the time of using breathing masks. The necessary therapeutic and preventive interventions regarding the complications of using masks, as well as planning to train personnel for the correct use of masks with minimal health effects are suggested.

**Supplementary Information:**

The online version contains supplementary material available at 10.1186/s12913-024-11000-4.

## Background

The COVID-19 pandemic has brought unprecedented challenges to healthcare systems worldwide, requiring Health Care Workers (HCWs) to adopt strict infection control measures to protect themselves [1]. Among these measures, the proper use of respiratory masks plays a crucial role in preventing the transmission of the virus [2]. Iran was among the initial countries impacted by COVID-19. In Iran, as in many other countries, HCWs have been at the forefront of the battle against COVID-19, facing various challenges in utilizing respiratory masks effectively [3]. Over 7.6 million Iranians have been infected by the SARS-CoV-2 virus, with more than 146,480 reported deaths as of August 2023 [4]. Amid the COVID-19 pandemic, Iran’s healthcare system experienced significant impacts as well [5].

Despite the passage of several years since the onset of the COVID-19 pandemic, new variant of the virus continues to emerge worldwide. It is crucial to be prepared for future pandemics and similar biological disasters.

Due to the SARS-CoV-2 virus transmission via respiratory droplets, the use of masks and personal protective equipment is essential [6]. The World Health Organization recommended the use of medical masks, such as surgical masks, for HCWs during the COVID-19 pandemic [7]. These masks are designed to provide a barrier to respiratory droplets and help reduce the transmission of the virus [8].

Few studies have been devoted to negative aspects of using respiratory masks in human being. The physiological and adverse effects of using PPE have been investigated in a systematic review study [9]. In another review study, of skin problems related to the use of respiratory masks were studied [10]. Also, in some studies, a significant relationship has been found between the time of using masks and the severity of the adverse effects of using masks [11]. In all the above studies, questionnaires have been used to check the prevalence of these adverse effects among HCWs.

Incorrect use of masks is considered as the unsafe behaviors of HCWs. In some studies, unsafe behaviors are defined as disobeying an accepted safe method while working with the capability of causing an accident [12]. Since the reasons for unsafe behavior are complex and multifaceted, their prevention requires a clear understanding of important and influential factors. In various studies about the prevalence of unsafe behaviors in work environments, several factors such as individual characteristics, psychological aspects, safety conditions, perceived risk, and stress have been introduced as effective factors in demonstrating the unsafe behaviors [12–14]. However, the findings are still unable to provide a deep understanding of the underlying causes and motivations contributing to unsafe behaviors.

In the present study, unsafe behaviors while using respiratory masks is defined as the behaviors that are seen by some HCWs, which reduce the effectiveness of respiratory masks due to improper placement on the face or hand contact with the mask [15]. Some researchers in their studies indicated that other unknown factors are also effective in the unsafe behaviors [14]. However, the findings are still unable to provide a deep understanding of the underlying causes and motivations contributing to unsafe behaviors. Qualitative studies are needed to answer these questions and determine its causes. Hence, the present study is aimed to explore the factors affecting the unsafe behavior of HCWs while using respiratory masks during the COVID-19 pandemic through a qualitative study.

## Methods

### Study design

This study was carried out using conventional qualitative content analysis (item 9 in COREQ checklist). The interviews explored HCWs’ experiences regarding factors affecting the unsafe behavior in using respiratory masks during covid-19 pandemic in Iran. This research adheres to the guidelines outlined in the Consolidated Criteria for Reporting Qualitative Research (COREQ).

### Settings

This study was conducted in government and non-government hospitals in Tehran, Mashhad and Rafsanjan that admitted patients with COVID-19 disease. The authors’ place of work and access to participants were important causes of choosing the settings. Moreover, these hospitals experienced a large amount of patients seeking healthcare during the Covid-19 pandemic. This study was performed between December 2021 and April 2022.

### Participants

In this study, interviews were performed with healthcare workers (HCWs) including nurses, physicians and hospital workers who had direct contact with patients that used masks for more than 4 h in each work shift. Also, participants frequently utilized surgical masks. Among them, few employed filter masks or a combination of both types. The inclusion criteria were people with experience of using respiratory masks for more than one year and the ability to express their experiences and point of views. The sole exclusion criterion of the current study was a lack of interest in further participation. The participants were selected using purposive sampling method (item 10 in COREQ checklist) in which the researcher selected the most informed people who could explain their experiences regarding the research topic [16]. The number of participants was determined based on the data saturation principle in which no new concepts were obtained. Data saturation was achieved after 24 interviews, and to ensure saturation, two more interviews were also performed. Finally, the total number of participants was 26 people (items 12–13 in COREQ checklist).

### Data gathering

Data collection was performed through in-depth face to face (item 11 in COREQ checklist) semi-structured interviews. The first author, who received training in qualitative research methods, conducted all the interviews (items 1–5 in COREQ checklist). The participants were presented with information about the research topic, objectives, and the researchers’ identities. The researcher thoroughly described the study procedure to those who consented to participate, and written informed consent was obtained from all participants (items 6–8 in COREQ checklist). The data was gathered in the workplace of the participants. Additionally, demographic data of the participants was documented (items 14–16 in COREQ checklist). At first, 5 unstructured interviews were done to extract the primary concept, and then, 21 semi-structured interviews were conducted using the interview guide. The interviews were done in a quiet and comfortable place. The interviews started with simple and general topics and were gradually directed to specific questions based on the answers. Some of the questions were: Based on your experience, what factors are effective in not using your mask safely?

New concepts were extracted from each interview, and this process continued until data saturation was reached. After obtaining permission from the participants to record the interviews, the implementation of the interviews was done immediately after the completion of each interview to increase the accuracy of the obtained data. The duration of the interviews was between 15 and 40 min (30 min on average). Field notes were made during or after the interview and transcripts were returned to participants for the comments and corrections (items 17–23 in COREQ checklist).

### Data analysis

Data analysis was done using the five-step content analysis approach of Graneheim and Lundman [17]. Immediately after conducting each interview, the recorded file of the interview was transcribed in Word software. The interview text was read several times and based on the research question, all the content related to the participants’ experiences were extracted in the form of meaning units. In addition, notes were written in the margins of the text and then, the abstracted meaning units were designated as the code. Subsequently, the compiled codes were categorized into subcategories according to similarities. This process was repeated for all transcribed interviews until the main categories were established. The whole data analysis process was carried out by the researchers. Direct quotes from the interviews included in the results section to elucidate the codes, categories, and themes. (items 24–32 in COREQ checklist).

### Trustworthiness

The strategies of transferability, dependability, credibility outlined by Lincoln and Guba were employed to achieve data trustworthiness [18]. Credibility and dependability were established through data triangulation approach, which involved interviews and field notes. Furthermore, peer check and member check were applied for ensuring credibility. To obtain member check, the transcribed interviews and codes were shared with some participants to receive their feedbacks. In the case of peer check, the research team and independent experts were verified the extracted codes and sub-categories. Data transferability and Confirmability were met through the detailed explanation of the research stages and process.

## Results

Women were 50% of all participants and the highest frequency of education was bachelor’s degree (*n* = 17). Furthermore, the highest amount of work experience was 22 years (Table 1).

**Table 1:** Demographic characteristics of the participants

No	Variables		N	%
1	Age (year)	27–37	16	61.5
		38–48	10	38.5
2	Work Experience (year)	1–11	14	53.8
		12–22	12	46.2
3	Gender	Male	13	50
		Female	13	50
4	Educational Level	Bachelor	17	65.3
		Master	7	26.9
		Medicine	2	7.6

In the present study, 689 initial codes were identified in the initial writing, and after removing duplicate codes and cleaning, the number of final codes included 132 codes. After reviewing and analyzing the data, the factors affecting the unsafe behavior of HCWs while using respiratory masks were divided into 3 main categories and 8 sub-categories (Table 2). Categories included discomfort and pain (four sub-categories of headache and dizziness, skin discomfort, respiratory discomfort, feeling hot and thirsty), negative effect on performance (four sub-categories of effect on physical function, effect on cognitive function, system function vision and hearing), and a negative effect on the mental state (two subcategories of anxiety and depression).

**Table 2:** Codes, subcategories and categories of the unsafe behavior of health care workers’ in using respiratory masks during COVID-19

Category	Subcategory	Selected Code
Pain and Discomfort	Skin disorders	• Feeling itchy • Feeling irritated • Scar on the face
	Respirational disorders	• Difficulty in breathing • Increased heart rate
	Feeling hot and thirsty	• Moisture inside the mask • Heat generation inside the mask • Feeling thirsty
	Unfitness of mask and individual face	• My mask is tight and puts pressure on my face. • The mask strap is tight or loose • Feeling headache • feeling dizzy • The pressure of the mask strap on the ears • Strap pressure on the back of the head
Effects on performance	Effect on physical performance	• Getting tired quickly • Bruised feeling in the body • Reducing the speed of doing physical activity
	Effect on cognitional activity	• Decreased concentration • Decrease in accuracy • Weakness in vision • Hearing loss • Increased cognitive errors
Effects on mental status	Anxiety	• Increased stress • Restlessness or feeling on edge • Difficulty concentrating • Shortness of breath
	Depression	• Reduced communication with others • Difficulty talking to others • Feeling bored

### Pain and discomfort

Some of the participants reported that the reason for improper and unsafe use of the mask is feeling pain and discomfort, and the reasons include the four subcategories of headache and dizziness, skin discomfort, respiratory discomfort, discomfort caused by heat and thirst.

#### Skin disorders

The side effects of the mask on the skin are of the important factors in this category. Thus, some participants, due effects of the mask to their skin, limited the use of the mask or did not use it correctly. Among the skin problems experienced by the participants were acne and skin sensitivities, which in some cases required drug treatments. The subcategory of skin sensitivities such as itching and burning was mentioned by more than 70% of the samples as the most important cause of discomfort.

“…I can’t help touching my mask. After half an hour when I put on the new mask, my face, especially my nose, starts to itch badly and I often have to blow my nose from under the mask or over the mask with my fingers, palm or the back of my hand…” (P1)

#### Respiratory disorders

Most of the participants in the study noted to problems such as difficulty in breathing, heart palpitations, carbon dioxide and unpleasant smell inside the mask as the most important respiratory problems. Therefore, it can be one of the important reasons for removing the mask and unsafe behavior in using the mask.

“… at any opportunity, I remove my mask to take a breath…” (P15)

#### Feeling hot and thirsty

Temperature discomfort, especially in long-term use and when people had to use two masks, was mentioned as an annoying factor.

“… the heat inside the mask bothers me a lot, I sweat and the mask gets wet… no matter how much water I drink, I still feel thirsty…” (P6)

#### Unfitness of mask with the individual’s face

Another important point extracted from the interviews was the importance of when to use the mask. In this way, as the time of using the mask increased, the person’s feeling of discomfort due to the mismatch between the belt and the mask increased, because the feeling of pressure and pain on the nose, behind the ears, and the face usually occurs several hours after wearing the mask. Several participants reported experiencing discomfort and headaches after wearing the mask. Although These headaches were often short-term and didn’t have long-term complications according to the participants’ reports, they could affect the work performance of HCWs and their behavior in the correct use of respiratory masks.

“…. After a while, the mask puts pressure on my nose and parts of my head and face. Sometimes I touch and move it unintentionally…” (P3)

“… if I don’t move the mask on my face, I get a headache because the mask strap puts pressure on my head and nose…” (P21)

### Effects on performance

The participants reported that wearing a mask for a long time is one of their important problems in performing their duties, and one of the main categories extracted from this study is the effects on performance, which includes the physical, cognitive, vision and hearing performance.

#### Effects on physical performance

The effect on the physical performance of HCWs had less effect on their unsafe behavior in using masks than other cases. But when masks were used for a long time and people were more physically tired, sometimes people removed the mask to increase their ability to perform physical work.

“…when I wear a mask, it becomes difficult for me to walk and do physical work, as if I am short of breath…” (P17)

#### Effects on cognitive function

It was the most frequent subcategory. Because when people feel uncomfortable, their attention decreases and part of the working memory is involved in feeling uncomfortable. Of course, it should be noted that many of the participants in the present study reported the decrease in alertness to be an effective factor in reducing their cognitive performance.

“…When I take off the mask, I can focus better on my work. Especially when I wear it in longer times, I get tired. Many times, I move the mask to finish my job faster…” (P8)

Based on the participants’ point of views, data perception (understanding information through the visual and auditory systems) decreases while using the mask. However, the negative effect of mask on the visual performance affects the unsafe behavior of the HCWs in the incorrect use of the mask and moving it on the face more than other cases. Most of the people who used glasses reported the steam condensation under the glasses as an important cause of discomfort and interference of the mask with their work duties.

“…Using glasses with a mask is really annoying. I have eye pain and burning, and there is always a fog in front of my eyes…” (P2)

### Effects on mental status

Among the other main categories extracted in this study is the effects on mental status, which includes the subcategories of depression and anxiety. The negative effect of the mask on the mental state unconsciously affects the person’s behavior in using the respiratory mask.

#### Anxiety

Some of the participants in this study reported feeling anxious while wearing the mask for various reasons. Therefore, they refuse to wear masks, although they have no justification for doing so. In many cases, the participants in this study expressed that during higher psychological stress, they suffer more from wearing masks and tend to wear them improperly.

“… Sometimes I distractedly take off my mask so that the other person hears my voice better. However, there are many patients, So I am afraid of getting infected. Sometimes I have to speak loudly and this makes me furious … I worry about making a mistake or misunderstanding the conversation, and …” (P4)

#### Depression

One of the most important factors mentioned as a cause of depression was harder communication with colleagues and patients while wearing a mask. This occurs by increasing the physical and mental workload and placing people in social isolation. In this situation, HCWs sometimes consciously take off their masks, so that they can communicate with each other more conveniently.

“…When I wear a mask, I get tired when talking to others. I prefer not to talk to my colleague. Sometimes I don’t pay attention, I take the mask down so they can understand me …” (P5)

## Discussion

To the best of our knowledge, this research is one of the first qualitative studies to extract the experiences of HCWs for explaining the factors affecting the unsafe behavior of HCWs in using respiratory masks during the COVID-19 pandemic in Iran. Although many reasons can cause the unsafe behavior of HCWs in the correct use of respiratory masks in the hospital, according to the present results, three main categories include discomfort and pain, effects on performance, effects on mental status. Skin and respiratory discomforts and the negative effect of the mask on cognitive functions are among the most important factors affecting the unsafe behavior of HCWs in the field of correct use of respiratory masks.

Based on the present study, the participants experienced discomfort and pain while using the mask, and this was one of the important factors of unsafe use of respiratory masks. Discomfort while wearing masks has been confirmed in several studies [19]. Additionally, in a similar study, researchers found that wearing face masks during the COVID-19 era heightens the discomfort experienced by HCWs [20]. Some studies have delved into these discomforts in greater detail. For example, the prevalence of skin disorders among HCWs using PPE during the COVID-19 pandemic was reported to be significant [21]. Some researchers also reported significant prevalence of respiratory disorders and headaches when using PPE [22]. The findings of a study suggested that a novel form of headache has emerged among HCWs when using a mask during the COVID-19 pandemic. Both exacerbation of existing headaches and the onset of new headaches have been observed to rise with mask usage, irrespective of the use duration [23]. In some studies, a significant percentage of people reported feeling thirsty and dehydrated after long-term use of respiratory masks [24]. Several studies reported disturbing rates of perspiration from prolonged use of respiratory masks [25–27]. A similar study reported that prolonged exposure to masks and protective gear, especially among HCWs, can lead to various issues such as acne, skin irritation, cognitive impairment, and headaches [28]. According to the results of the present study, discomfort often causes HCWs to move the mask and disturb the correct fitness of the mask on their face.

The results of the present study indicated that respiratory masks have the ability to hinder the work performance of their users. Various studies have confirmed the adverse effect of respiratory masks on HCWs performance. A similar research indicated that respiratory masks reduce physical performance [29]. Several studies have highlighted the issue of mask users’ ability to see and read being hindered by fogging of glasses [22, 27, 30]. The feel of weakness to perform cognitive tasks has also been reported in various studies [31, 32]. An increase in physical fatigue has been mentioned in some studies as an adverse effect of respiratory masks [27, 31]. A research showed the effect of respiratory mask on hearing and visual performance [33]. Another study reported that high-protection respiratory masks reduced physiological and psychological ability, especially if the workers perform physical work [34].

The third category is related to the negative impact on the psychological state of HCWs. Some studies noted the use of some PPE, including respiratory masks, as one of the possible reasons for the increase of mental health problems among HCWs [35, 36]. Before the prevalence of the COVID-19 virus, the hypothesis of the negative effect of respiratory masks on the mental state of people was investigated and confirmed by some studies [37]. Furthermore, one study reported that wearing respiratory masks leads to an increase in anxiety [38].

The non-ergonomic nature of respiratory masks (the lack of suitability of masks for people for long-term use) can affect the effectiveness of respiratory masks by encouraging people to perform unsafe behaviors in using respiratory masks [39]. An important point was that the attitude and knowledge of health care works regarding the use of respiratory masks were not identified as the cause of unsafe behavior of HCWs. However, this factor has been reported in some previous studies as a reason for people not using PPE properly [40]. The COVID-19 pandemic situation and the extensive information collected about this pandemic may improve the level of awareness and the attitude of the HCWs.

The escalation in infection rates among HCWs, despite receiving training and utilizing personal protective equipment, served as a catalyst for this research endeavor. So far, there has been a deficiency in the context-specific research that could offer a more profound understanding of this issue. Therefore, the outcomes of this qualitative study may prove beneficial in enhancing the design and execution of respiratory protection programs for HCWs in infectious hospital departments or during similar pandemics.

### Implications for nursing practice

It is expected that the findings of this study can provide a better understanding of the factors influencing the unsafe behavior of HCWs while using masks. Furthermore, it can be used as a preliminary study to evaluate the effectiveness of safety and infection control programs in hospitals in the COVID-19 pandemic and similar disasters in the future.

## Conclusion

Discomfort and pain, effects on performance, and effects on mental status are important factors for unsafe behavior of HCWs’ in using respiratory masks. Our results could contribute to the identification and analysis of possible scenarios to reduce unsafe behaviors in the use of respiratory masks. Accordingly, it is recommended to provide the necessary therapeutic and preventive interventions regarding the complications of using masks. Planning to reduce the side effects of masks and training personnel on the correct use of masks with minimal health effects are recommended as well.

### Limitations

The physical and cognitive workload of HCWs which increased during the COVID-19 pandemic [41], had possible impacts on the work ability of the staff [42]. Therefore, their explanation about the negative effects of wearing masks may be affected by their specific working conditions.

### Electronic supplementary material

Below is the link to the electronic supplementary material.

Supplementary Material 1

Supplementary Material 2

## Data Availability

The datasets used during the current study are available from the corresponding author on reasonable request.
