# Supplementary material for: Exploring factors affecting the unsafe behavior of health care workers’ in using respiratory masks during COVID-19 pandemic in Iran: a qualitative study
Source: BMC Health Serv Res. 2024 May 9;24:608. doi: 10.1186/s12913-024-11000-4 (PMC11080203; doi:10.1186/s12913-024-11000-4)
Supplement: Supplementary file 1 — Supplementary Material 1 [file 12913_2024_11000_MOESM1_ESM.docx]

**Interview guide**

**Research title: " Exploring Factors Affecting the Unsafe Behavior of Health care Workers’ in Using Respiratory Masks During COVID-19 Pandemic in Iran"**

The name of the interviewer: Azadeh Tahernezhad, PhD student of health in disasters and emergencies, School of Public Health and Safety, SBMU, Tehran, Iran.

**The purpose of the research**

this research aims to explore factors affecting the unsafe behavior of health care workers’ in using respiratory masks during covid-19 pandemic in Iran".

**The nature of the interview**

The interview is conducted in order to receive and use the experiences of healthcare workers regarding the human factors affecting the incorrect use of respiratory masks. Obviously, there are no right or wrong answers in this interview, but the researcher only seeks to know the perceptions and experiences of mask users in this field. It is expected that the interview will last about 20-40 minutes. If the interview is not completed in the mentioned time, the interviewee is requested to add some time to the interview if there is enough time.

**Keeping confidential documents**

All the interviewees are assured that the confidentiality of the answers will be fully respected and the data will be used only in line with the goals of the research and only the researcher will have access to the information. In any case, under no circumstances the interviewee's name won’t be included anywhere.

For the purpose of more in-depth investigation and analysis, with the knowledge and consent of the interviewee, a voice recorder will be used to record the voice. It is worth mentioning that the data extracted from interviews are considered completely confidential and can only be identified by a code or number.

**voice recording**

Is it possible for me to get help from recording this interview?

Do you have any questions before the interview?

- Turn on the recorder after the participant's agreement

**1- Your professional experience**

I would like to know your background and experiences before discussing the main subjects under investigation.

• How much do you know about the effect of using personal protective equipment in the prevention of COVID-19?

• How many years of work experience do you have and what is your educational and work background?

• How much have you been trained for using a breathing mask correctly and know about the factors affecting it?

**2- The main topics and questions of the interview**

2-1- What are your experiences about the use of respiratory masks during the COVID-19 pandemic?

2-5 What obstacles cause you to use the breathing mask incorrectly?

2-6 What suggestions do you have to remove the obstacles to the incorrect use of breathing masks?

In order to probing and deep investigation of people's experiences to reach saturation, the researcher uses how and why? Who or what questions. Furthermore, questions are formed during the interview to maintain the dynamics of the interview and guide it toward the interview goals.
